# *GsMYB10* encoding a MYB-CC transcription factor enhances the tolerance to acidic aluminum stress in soybean

**DOI:** 10.1186/s12870-024-06004-5

**Published:** 2024-12-26

**Authors:** Ce Yang, Xiang Lu, Dan Du, Zhongyi Liang, Cheng Li, Kang Hu, Hongjie Wang, Yanbo Cheng, Tengxiang Lian, Hai Nian, Qibin Ma

**Affiliations:** 1 The State Key Laboratory for Conservation and Utilization of Subtropical Agro-bioresources, South China Agricultural University, Guangzhou, Guangdong 510642 China; 2 Key Laboratory of Plant Molecular Breeding of Guangdong Province, College of Agriculture, South China Agricultural University, Guangzhou, Guangdong 510642 China; 3 Guangdong Subcenter of the National Center for Soybean Improvement, College of Agriculture, South China Agricultural University, Guangzhou, Guangdong 510642 China; 4 Guangdong Provincial Laboratory of Lingnan Modern Agricultural Science and Technology, South China Agricultural University, Guangzhou, Guangdong 510642 China; 5 Zengcheng Teaching and Research Bases, South China Agricultural University, Guangzhou, Guangdong 510642 China; 6 College of Agriculture, South China Agricultural University, Guangzhou, Guangdong 510642 China

**Keywords:** Soybean, Aluminum stress, MYB-CC family, Transcription factor

## Abstract

**Background:**

MYB transcription factors (TFs) play crucial roles in the response to diverse abiotic and biotic stress factors in plants. In this study, the *GsMYB10* gene encoding a MYB-CC transcription factor was cloned from wild soybean BW69 line. However, there is less report on the aluminum (Al)-tolerant gene in this subfamily.

**Results:**

The *GsMYB10* gene was up-regulated by acidic aluminum stress and rich in the roots with a constitutive expression pattern in soybean. It was found that GsMYB10 protein contains the MYB and coiled-coil (CC) domains, localizes in the nucleus and holds transcriptional activity. The analysis of the transgenic phenotype revealed that the taproot length and root fresh weights of the *GsMYB10*-OE plants were greater than those of the wild type when subjected to AlCl_3_ treatments. While the accumulation of Al^3+^ in root tip of *GsMYB10* transgenic plants (59.37 ± 3.59 µg/g) significantly reduced compared with that of wild type (80.40 ± 3.16 µg/g) which were shallowly stained by hematoxylin under the treatments of AlCl_3_. Physiological indexes showed that the proline content significantly increased 39–45% and the malondialdehyde content significantly reduced 37–42% in *GsMYB10-OE* plants compared with that of wild type. Transcriptomic analysis showed that overexpression of *GsMYB10* induced a large number of differentially expressed genes (DEGs) with Al-treatment, which were related to wall modification related genes included PGs (such as *Glyma.19g006200*, *Glyma.05g005800*), XTHs (such as *Glyma.12g080100*, *Glyma.12g101800*, *Glyma.08g093900* and *Glyma.13g322500*), NRAMPs and ABCs.

**Conclusions:**

In summary, the data presented in this paper indicate that *GsMYB10*, as a new soybean MYB-CC TF, is a positive regulator and increases the adaptability of soybeans to acidic aluminum stress. The findings will contribute to the understanding of soybean response to acidic aluminum stress.

**Supplementary Information:**

The online version contains supplementary material available at 10.1186/s12870-024-06004-5.

## Background

Aluminum (Al) toxicity is a primary factor reducing crop yields on acidic soils, as much as 40–50% of the world’s arable land is acidic [1]. At low soil pH values (pH < 5.0), the phytotoxic Al^3+^ is released from the soil, which can rapidly inhibit root growth and damage physiological functions, thus reduce crop yield [2]. Therefore, enhancing the aluminum tolerance of crops is one of the effective ways to improve crop yield on acidic soil.

Many plant species have developed strategies to cope with Al toxic tolerance mechanism and recovery from Al-induced damages. Two main types of Al resistance mechanisms have been documented. The organic acid anions are secreted through the roots by chelating Al^3+^ ions and preventing them from entering the roots in Al exclusion mechanism. The Al tolerance mechanisms are classified as apoplasmic or symplasmicin mechanisms which are relative to property modification of the root cell wall, and sequestration or compartmentalization of Al once it enters the root symplast [3, 4]. In plants, the first identified aluminum resistance genes were malate and citric acid efflux transporters encoding ALMT (aluminum activated malate transporters) and MATE (multidrug and toxic compound extrusion) family membrane transporters, respectively [5]. Subsequently, more genes related to aluminum resistance have been reported. A large number of *MATE* members were implicated in citric acid efflux, such as *AtMATE* in *Arabidopsis thaliana* [6], *TaMATE1* in wheat (*Triticum aestivum*) [7], *OsFRDL4* in rice (*Oryza sativa*) [8], *ZmMATE1* and *ZmMATE2* in maize (*Zea mays*) [9]. ALMT family is control of the Al tolerance mechanism based on malate exudation. *TaALMT1* is the first Al-tolerant gene cloned in plants, which encodes a transporter protein that involves the isolation of malate from the root tip and is responsible for wheat tolerance to aluminum stress [5]. Functional ALMT homologs associated with Al tolerance were also identified in *Arabidopsis* (*AtALMT1*) [10], *Secale cereale* (*ScALMT1*) [11], and *Hordeum vulgare* (*HvALMT1*) [12]. In addition, plants improve aluminum resistance by altering Al^3+^ binding in cell wall through cell wall components modification. The most important effect of aluminum is on cell wall enzymes among the cell wall modification, including pectin methylesterase, endo-β-1,4-glucanases, xyloglucan endotransglucosylase/hydrolases (XTHs), and expansins. For example, pectin methylation in root tip cell wall was related to aluminum resistance in *Arabidopsis* and rice, *XTH31* affects Al sensitivity by modulating cell wall xyloglucan content and Al binding capacity in *Arabidopsis* [13].

Transcriptional responses of plants to environmental stresses have been extensively investigated and TFs regulate the expression of genes at the transcriptional level. However, only a few transcription factors regulating Al tolerance have been identified in several higher plant species. Two zinc-finger proteins, sensitive to proton rhizotoxicity 1 (*STOP1*) in *Arabidopsis* and Al^3+^ resistance transcription factor 1 (*ART1*) in rice were conferred hypersensitivity to Al toxicity [14, 15]. Furthermore, the expression of many Al-tolerance genes (e.g. *MATE*, *ALS1*, *FRDL4*,* FRDL2*,* ALMT1*) are regulated by these two transcription factors [16–18]. Two WRKY family members *AtWRKY46* and *OsWRKY22* also have mechanisms related to aluminum resistance. *AtWRKY46* is a negative regulator of *ALMT1*, *wrky46* leads to increased malate secretion and reduced Al accumulation in root apices, and thus confers higher Al resistance [19]. *OsWRKY22* promotes Al-induced increases in *OsFRDL4* expression by enhancing Al-induced citrate secretion and Al tolerance in rice [20]. *GmWRKY21*, a soybean WRKY transcription factor gene, enhances the tolerance to aluminum stress in *Arabidopsis thaliana* [21].

MYB transcription factors play a crucial role in regulating plant responses to abiotic stresses [22], including drought, salinity, cold, and aluminum toxicity. For instance, expression of the *Arabidopsis AtMYB44* gene confers drought/salt-stress tolerance in transgenic soybean [23]. *GhMYB36* positively regulated drought stress response both in *Arabidopsis* and cotton (*Gossypium hirsutum*) [24]. *GmMYB68*-overexpression lines showed enhanced resistance to salt and alkali stresses [25]. Transgenic apple calli and Arabidopsis with overexpression of *MdMYB23* exhibited increased cold tolerance [26]. Furthermore, *MsMYB741* enhances the accumulation of flavonoids in the root system and promotes root tip secretion by transcriptionally activating the expression of *MsPAL1* and *MsCHI*, thereby improving alfalfa’s resistance to aluminum stress [27]. *OsMYB30* negatively regulates Al resistance in rice [28]. *GsMYB7* may enhance soybean tolerance to acidic aluminum stress by regulating the downstream genes [29]. MYB-CC TFs are the members of the MYB TF superfamily which are characterized by containing a conserved MYB DNA-binding domain and a coiled-coil (CC) domain [30]. The MYB-CC transcription factor has been demonstrated to play a role in the inorganic phosphate (Pi) starvation response and regulates a series of Pi starvation-inducible genes, such as, *AtPHR1s* [31]. The genes including *PHL1*, *PHL2*, *PHL3* (UNE16) [32], *OsPHR1*, *OsPHR3* [33], *OsPHR2* [34], *OsPHR4* [35], *GmPHR1* [36], and *GmPHR25* [37] respond to phosphorus starvation. However, there is limited information regarding aluminum (Al)-tolerant genes within this subfamily.

Soybean is one of the most important crops that serve as a crucial source of high-quality protein foods and vegetable oils. Nevertheless, acidic aluminum stress has inflicted severe consequences on soybean yields, particularly in the southern regions. In contrast, wild soybean (*Glycine soja*) has undergone long-term natural selection and has evolved special mechanisms to survive in aluminum stress. Previous studies have shown that the *GsMYB10* gene is rapidly induced in response to aluminum stress in the wild soybean BW69 line (an aluminum tolerant line of *Glycine soja*) [38]. In this study, the *GsMYB10* gene encoding a MYB-CC transcription factor was cloned from the BW69 line and transformed in recipient plant Huachun6. The *GsMYB10* gene could improve the tolerance of transgenic plants to aluminum stress. The results revealed that *GsMYB10* was involved in plant aluminum stress response, which will provide more genetic resources for soybean molecular breeding resistant to aluminum toxicity.

## Results

### Isolation and sequence analysis of *GsMYB10*

**Fig. 1:**
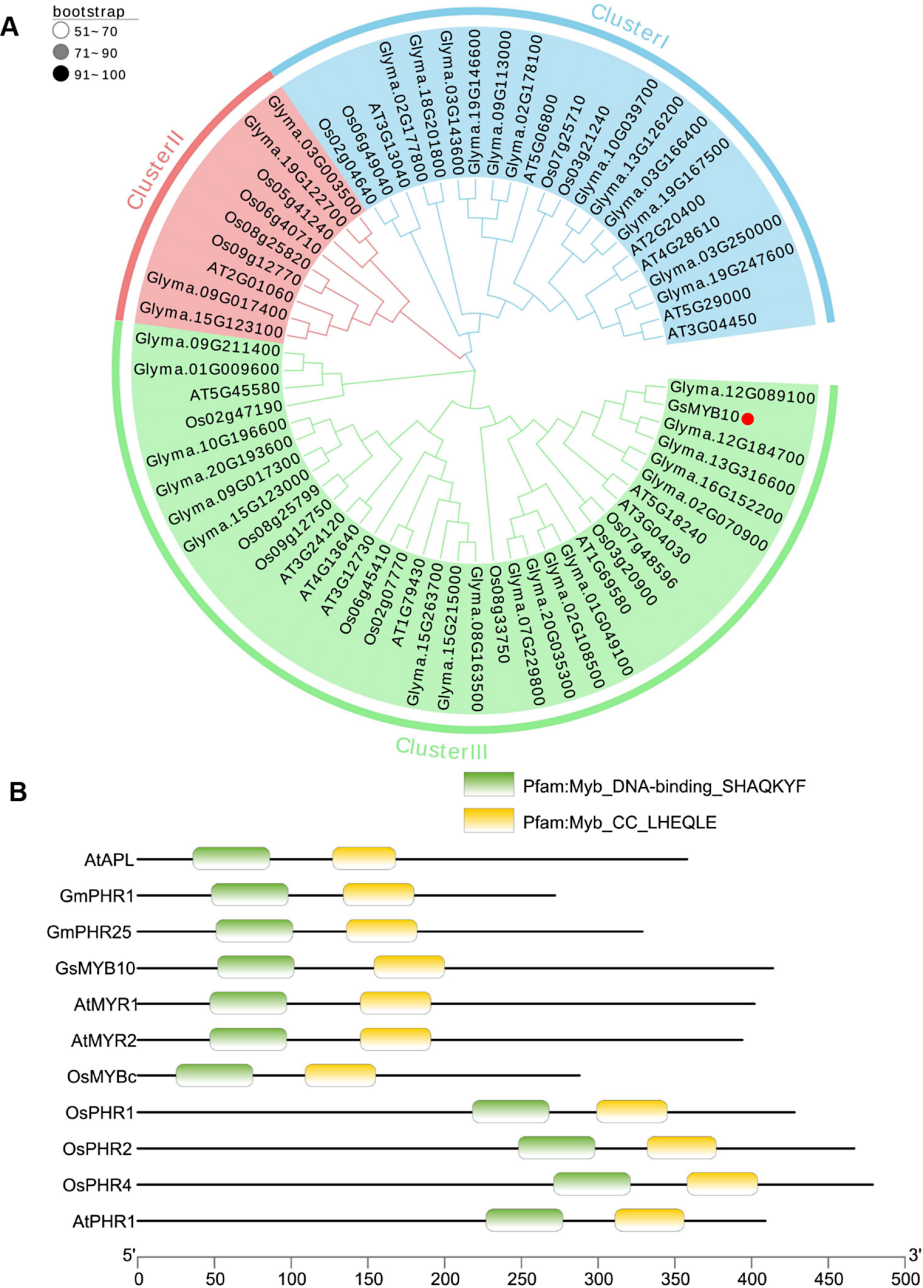
The phylogenetic tree of MYB-CC family proteins from soybean, rice and *Arabidopsis*. (**A**) The phylogenetic tree was constructed using MEGA X with the Maximum likelihood (ML) method. The three groups corresponding to three branches are marked by numbers (I-III). Bootstrap values in percentages (1000 replicates) are indicated on the nodes. Different subgroups use different colors of clades to distinguish. B and C are comparative alignments of conserved domain sequences of known functional genes in the MYB-CC family. (**B**) The MYB domain and CC (coiled-coil) domain of GsMYB10 and other MYB-CC proteins. The Genbank accession numbers of proteins or genes loci for other species are as follows: *AtAPL* (At1g79430), *GmPHR1* (Glyma.19g122700), *GmPHR25* (Glyma.15g123100), *AtMYR1* (At5g18240), *AtMYR2* (At3g04030), *OsMYBc* (LOC_Os09g12770), *OsPHR1* (LOC_Os03g21240), *OsPHR2* (LOC_Os07g25710), *OsPHR3* (LOC_Os02g04640), *OsPHR4* (LOC_Os06g49040), *AtPHR1* (At4g28610), *AtUNE16* (At4g13640)

The full-length genome sequence of *GsMYB10* is 3695 bp including 6 exons and 5 introns, containing an open reading frame of 1245 bp. The full length cDNA of *GsMYB10* was cloned from the wild soybean BW69, which encoded the same sequence with cultivar soybean Huachun 6. To explore the phylogenetic associations among the identified genes in different MYB-CC gene families, we aligned 35 soybean, 15 *Arabidopsis*, and 16 rice MYB-CC proteins and constructed phylogenetic trees using the maximum likelihood method (Fig. 1A). Phylogenetic analysis showed that the MYB-CC family could be classified into three clusters (from I to III). The GsMYB10 protein belongs to class III, shares a close evolutionary relationship with MYR1 (AT5G18240) and MYR2 (AT3G04030) in *Arabidopsis*. In addition, the analysis of conserved motif distributions revealed that the GsMYB10 protein has a conserved MYB DNA binding domain and a coiled-coil (CC) domain with other published MYB-CC proteins (Fig. 1B).

### Expression patterns of *GsMYB10* in tissues and response to Al stress

**Fig. 2:**
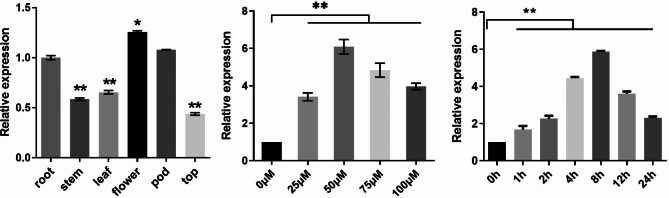
Expression patterns analysis of *GsMYB10* in wild soybean BW69 line. (**A**) Tissue expression pattern of *GsMYB10*. The samples of roots, stems, leaves, flowers, pods and tops (apical tissues) were harvested during the pod stage. (**B**) The three-day seedlings were cultured in 0.5 mM CaCl_2_ solution containing 50 µM AlCl_3_ (pH 4.5) for 0, 1, 2, 4, 8, 12 and 24 h. (**C**) Soybean seedlings were treated with 0, 25, 50, 75 and 100 µM AlCl_3_ in 0.5 mM CaCl_2_ solution (pH4.5) for 8 h. Total RNA was extracted from root apices (0–6 cm). The data were represented as the mean ± SD of three biologic replicates. Student’s *t*-test was used to calculate the *p*-values, *, *P*<0.05;**, *P <* 0.01

The transcript levels of *GsMYB10* in BW69 and Huachun6 plants were determined by Quantitative real-time PCR (qRT-PCR) analysis of roots, stems leaves, flowers, pods, and tops. The results showed that *GsMYB10* was constitutively expressed and was highly expressed in roots. In addition, *GsMYB10* was also expressed in high abundance in flowers and fruit pods of wild soybean BW69 plants (Fig. 2A and Supplementary Fig. 1A). In addition, *GsMYB10* responded to acid-aluminum stress at different concentrations. The transcripts of *GsMYB10* were the highest in BW69 with 50 µM Al-treatment (Fig. 2B), meanwhile, were the highest in Huachun6 under 75 µM Al-treatment (Supplementary Fig. 1B). The expression of *GsMYB10* was up-regulated by acidic aluminum with the treatment time in the Huchun6. However, the expression levels of *GsMYB10* in BW69 showed a trend of increasing first and then slowly decreasing with the treatment time. The transcript of *GsMYB10* in BW69 was up to the highest level at the AlCl_3_ treatment of 8 h (Fig. 2C and Supplementary Fig. 1C).

### Transcriptional activation activity assay and subcellular localization

**Fig. 3:**
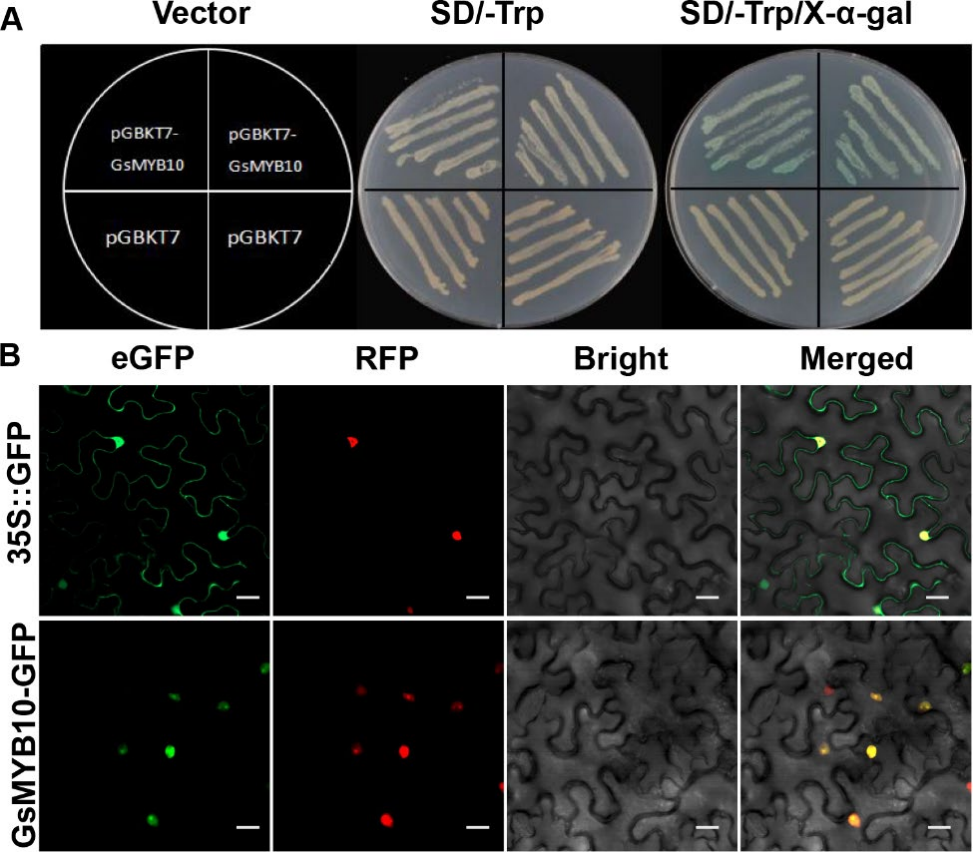
Transcriptional activity assays and subcellular localization of GsMYB10 protein. (**A**) Transcriptional activation activity of GsMYB10 protein in yeast cells. (**B**) Subcellular localization of the 35S::GsMYB10-GFP fusion protein in leaf epidermal cells of *Nicotiana benthamiana*. Leaf epidermal cells transformed with 35S::GFP were used as a control. The RFP was nuclear localization protein marker (PJIT-mCherry-Nuc). Scale bars = 10 μm

To verify whether the GsMYB10 protein has transcription activity, the plasmid pGBKT7 (control) and the fusion plasmid pGBKT7-GsMYB10 were transformed into yeast Y2H gold, respectively. The yeast cells were grown on control medium plates (SD/-Trp) or selective medium plates (SD/-Trp/-X-α-Gal). The Y2H strains containing pGBKT7-GsMYB10 grew and showed blue color on selective medium (Fig. 3A). The results suggested that GsMYB10 protein has transcriptional activation activity.

To detect the subcellular localization of the GsMYB10 protein, both 35S-GFP and 35S-GsMYB10-GFP plasmids were transfected into the young leaves of tobacco plants with the nuclear localization protein marker (PJIT-mCherry-Nuc), respectively. The analysis using laser confocal microscopy showed that 35S-GFP was distributed throughout the whole cells, whereas the 35S-GsMYB10-GFP fusion protein localizes only in the nucleus (Fig. 3B). The results indicated that GsMYB10 is a nuclear localization protein.

### *GsMYB10* improved the tolerance of the overexpressing lines to Al-stress

**Fig. 4:**
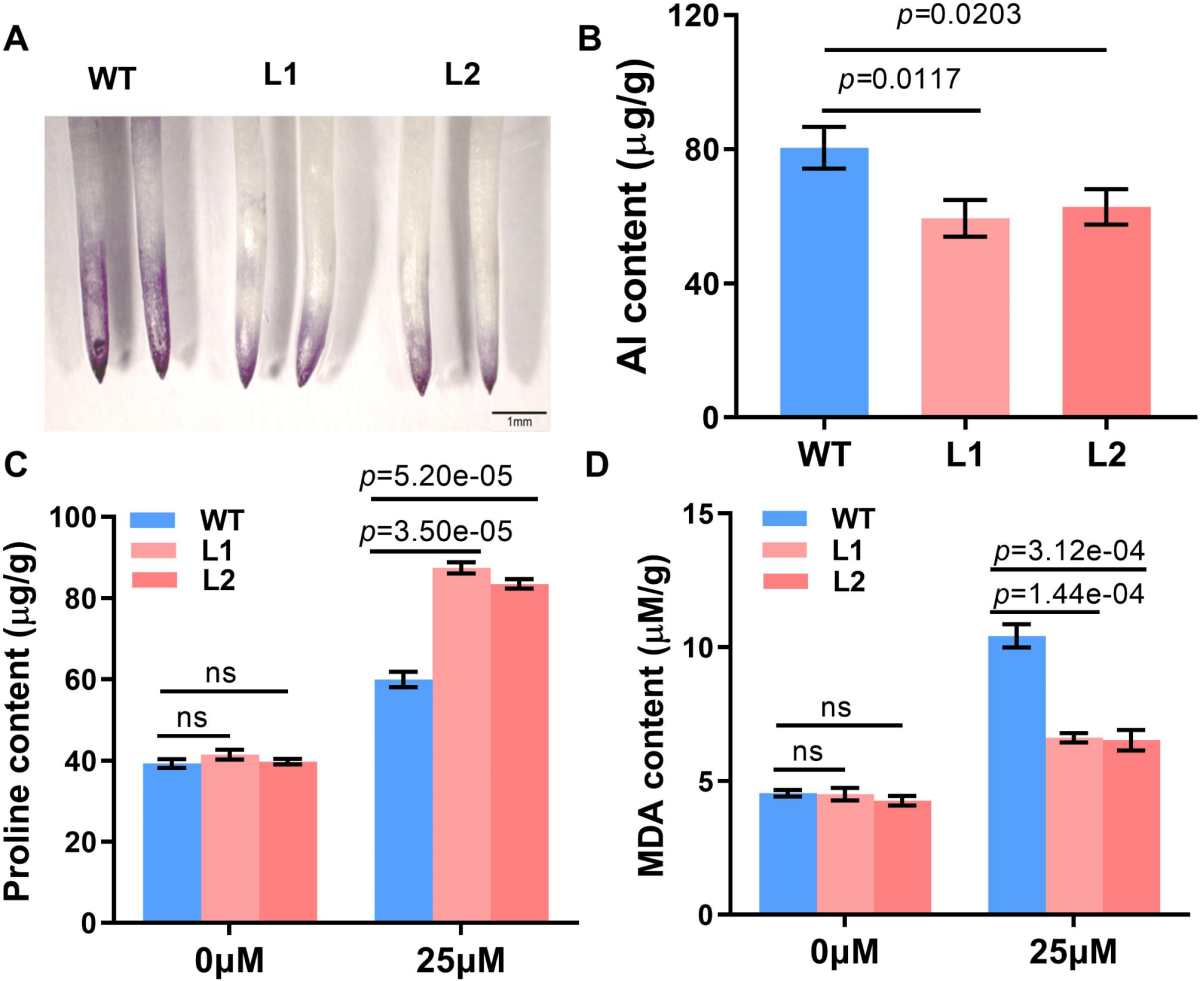
Physiological indexes related to aluminum stress change. The hematoxylin staining (**A**) root Al content (**B**) proline concentration (**C**) and MDA concentration (**D**) in transgenic plants and wild type under Al stress. Both WT and *GsMYB10* transgenic lines were exposed to 0.5 mM CaCl_2_ solution with 25 µM AlCl_3_ (pH 4.5) for 8 h (in **A** and **B**) or 24 h (in **C** and **D**). Data were represented as mean ± SD of three biological replicates. Student’s *t*-test was used to calculate the *p*-values, ns = no significant difference

More than 8 transgenic lines were obtained by transforming *GsMYB10* in Huachun 6. Three lines of T_3_ generations with high expression levels were selected for the phenotype identification (Supplementary Fig. 2). Hematoxylin staining is an Al indicator in assessment of Al localization and accumulation in roots tip. *GsMYB10-OE* plants and wild type were stained with hematoxylin after treatment in a solution consisting of 25 µM AlCl_3_ for 8 h to verify the aluminum resistance. The results showed that the roots of *GsMYB10* transgenic lines were lightly stained compared with those of wild type (Fig. 4A). The content of aluminum ions in root tips of transgenic plants was 59.37 ± 3.59 µg/g, which was lower than that of wild type 80.40 ± 3.16 µg/g (Fig. 4B). These results indicated that *GsMYB10* enhances resistance to aluminum toxicity by reducing the accumulation of aluminum.

The phenotype of short-term (24 h) Al treatment was first performed to identify the phenotypes as shown in Supplementary Fig. 3. The taproot relative elongation of transgenic lines was higher than that of the wild type treated with different Al concentrations. In addition, MDA is one of the most important products of membrane lipid peroxidation, and its production can also aggravate the damage of membrane. Al treatment significantly increased the accumulation of MDA. The content of MDA in root of transgenic lines was 6.57 ± 0.27 µM/g lower than that of WT 10.42 ± 0.43µM/g (Fig. 4C). Overexpression of *GsMYB10* contributes to reducing MDA levels under aluminum stress and reduces Al toxicity damage in plant roots. Proline plays an important role in protecting plants against various abiotic stresses. The proline content of the transgenic plants and wild-type plants increased after aluminum treatment, but the proline content in the *GsMYB10-OE* plants was much higher than that in the wild-type plants (Fig. 4D).

**Fig. 5:**
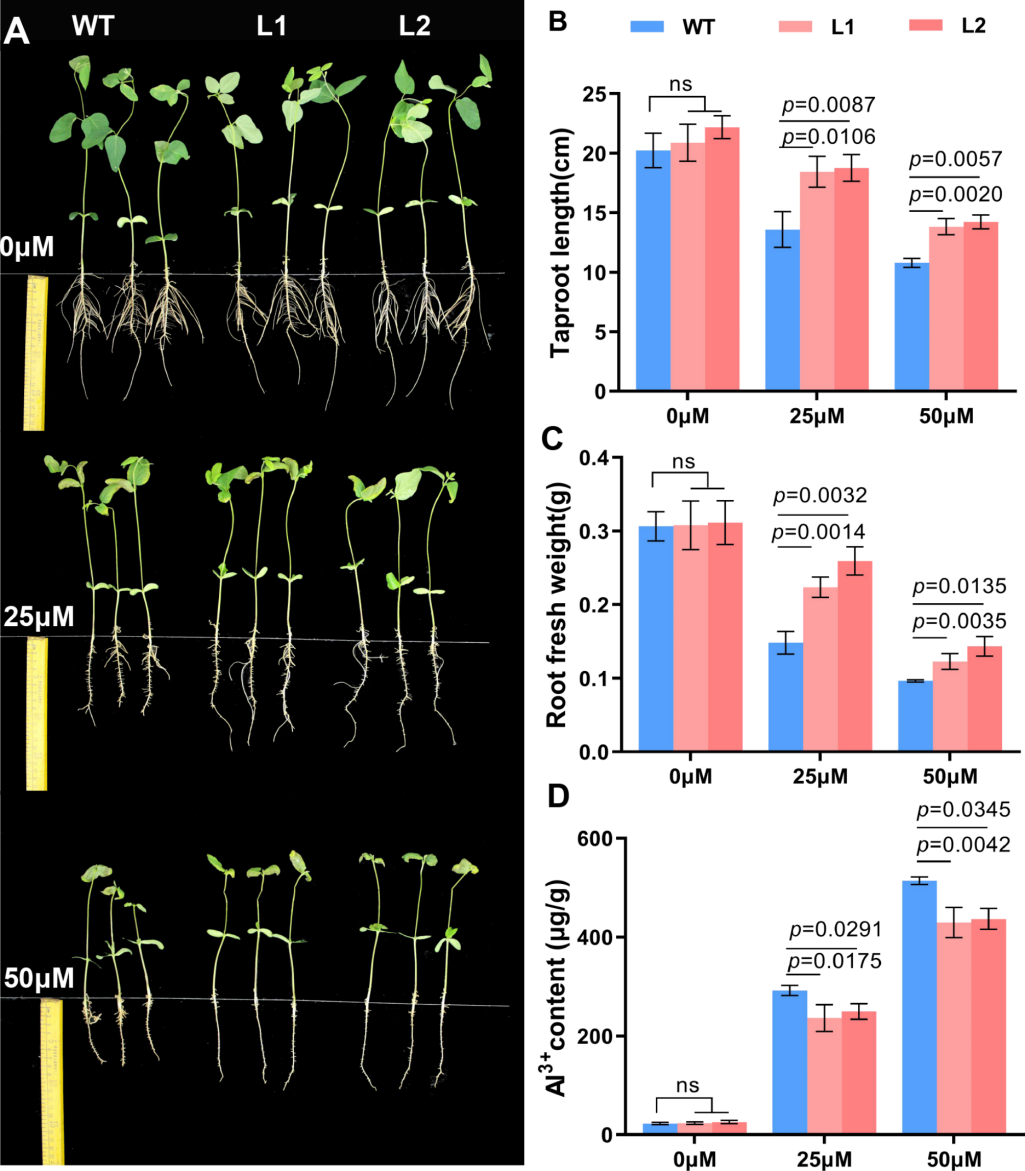
Overexpression of *GsMYB10* conferred enhanced aluminum tolerance in transgenic plants. (**A**) The phenotype of *GsMYB10* and wild type soybean seedlings which were treated using the solution including 0 µM, 25 µM and 50 µM AlCl_3_ in 0.5 mM CaCl_2_ solution (pH4.5) for 7 days. The taproot length (**B**), the fresh weight (**C**) and the aluminum content (**D**) of the underground part of transgenic lines (L1, L2) and wild type (WT). Data were represented as mean ± SD of three biological replicates, Student’s *t*-test was used to calculate the *p*-values, ns = no significant difference

Subsequently, we performed phenotypic identification at seedling stage (two true leaves open). The results show that the *GsMYB1*0-OE plants (L1, L2) were phenotypically identical to wild-type (WT) plants without Al treatment. However, the growth of primary and lateral roots of the plants was significantly inhibited under 25 µM and 50 µM aluminum treatments (Fig. 5A). Under the treatments of 25 µΜ and 50 µΜ AlCl_3_, the taproot lengths (Fig. 5B) and the root fresh weights (Fig. 5C) of *GsMYB10-*OE plants were significantly higher than wild type, and the root tip aluminum ion content in *GsMYB10-*OE plants was significantly lower than that of wild-type plants (Fig. 5D). Taken together, these results showed that *GsMYB10* overexpression enhance the soybean tolerance to aluminum stress.

### Transcriptome analysis of *GsMYB10* transgenic soybeans

**Fig. 6:**
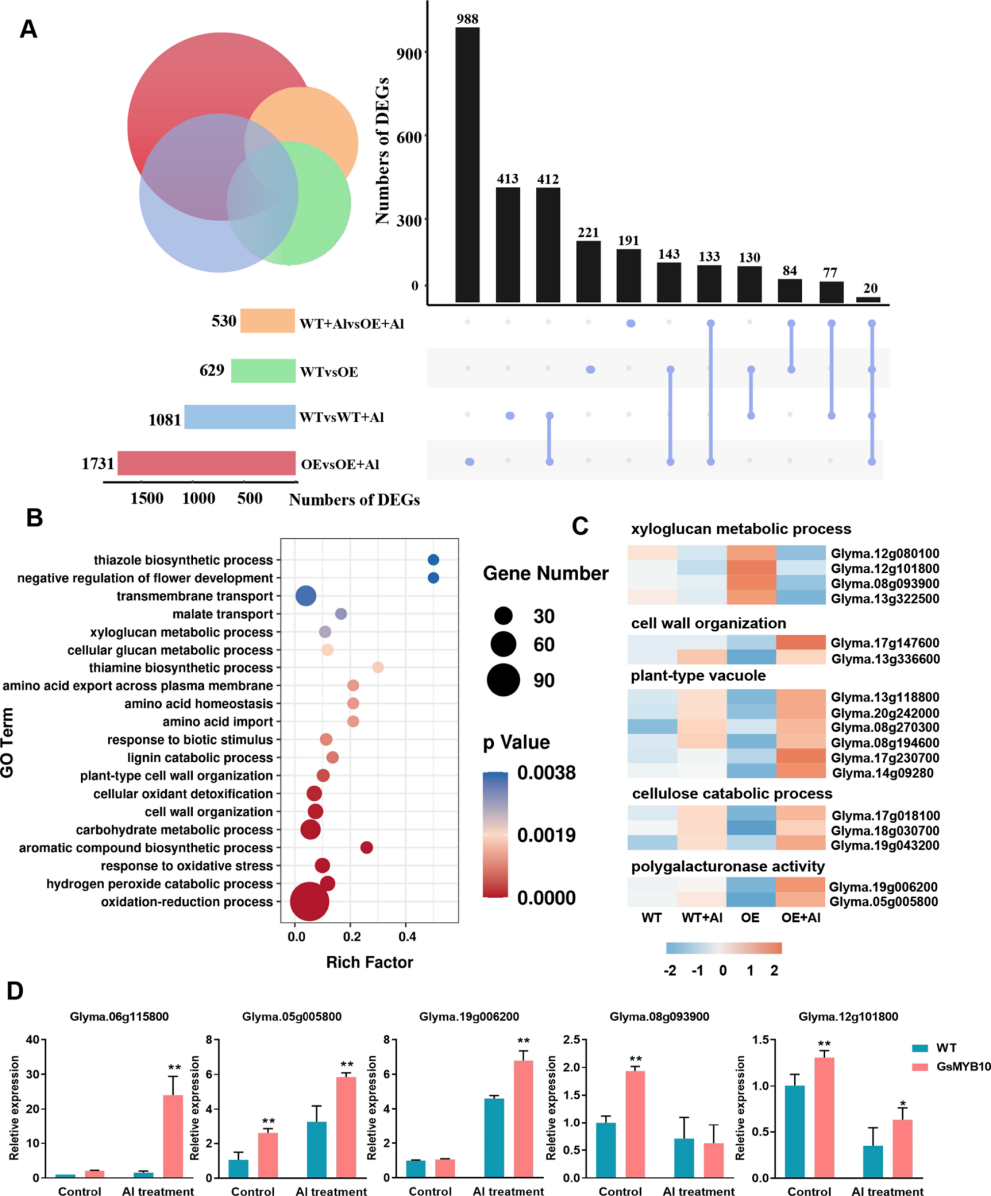
Transcriptome analysis of differentially expressed genes regulated by *GsMYB10*. (**A**) Upset diagram showing number of differentially expressed genes (DEGs) in different groups. (**B**) GO terms which were statistically enriched in *GsMYB10* regulated genes involved in aluminum treatment which were identified using the DEGs in A. (**C**) Heatmap of DEGs in the GO terms of xyloglucan metabolic process, extracellular region, plant-type vacuole, cellulose catabolic process, polygalacturonase activity. (**D**) Expression levels of the Al-responsive genes in *GsMYB10* overexpression transgenic soybean plants. Data were represented as mean ± SD of three biological replicates. Student’s *t*-test was used to calculate the *p*-values, *, *P <* 0.05; **, *P <* 0.01

To investigate the role of *GsMYB10* in response to aluminum stress, RNA sequencing (RNA-seq) was conducted using the *GsMYB10-OE* plants (L1) and wild-type plants under control conditions and aluminum treatment (25 µM AlCl_3_ for 8 h). The analysis of RNA-seq results showed that 629 genes were identified as differentially expressed genes (DEGs) in the *GsMYB10-OE* compared to the wild type (WTvsOE). Following the 8 h aluminum treatment, there were 530 DEGs identified in the comparison of WT + Al and OE + Al. Meanwhile, 1081 genes were differentially expressed in the wild type relative to the control treatment (WTvsWT + Al), with 822 upregulated genes and 259 downregulated genes (Fig. 6A and Supplementary Fig. 4). However, more transcriptomic changes in *GsMYB10-OE* plants (OEvsOE + Al) were due to aluminum treatment relative to the wild-type plants (WTvsWT + Al). The results suggested that overexpression *GsMYB10* regulated the expression of a large number of genes under aluminum stress.

To investigate the biological processes in which *GsMYB10* participates under aluminum stress, Gene Ontology (GO) enrichment analysis was performed using the 988 DEGs in comparisons (OEvsOE + Al) and the 133 DEGs (only shared by OEvsOE + Al and WT + AlvsOE + Al) (Fig. 6A). GO terms were especially enriched in the processes including oxidation-reduction process, hydrogen peroxide catabolic process, cell wall organization, cellular glucan metabolic process, xyloglucan metabolic process (Fig. 6B). The cell wall has an extremely important role in resisting acid-aluminum stress, acting as a fixation function for Al^3+^ and improving the aluminum tolerance of plants. Intriguingly, the transcript levels of cell wall related genes were dramatically changed in *GsMYB10-OE* plants, especially upon aluminum treatment (Fig. 6C). Furthermore, the qRT-PCR was carried out to confirm the results of transcriptome analysis. The tested DEGs were regulated under aluminum treatment, which was consistent with those of transcriptome analysis (Fig. 6D and Supplementary Fig. 5). The results suggested that cell wall related genes may contribute to enhancing aluminum resistance in *GsMYB10-OE* plants.

## Discussion

Aluminum toxicity in acidic soil is one of the major constraints for crop production worldwide. Searching for novel genes involved in Al-tolerance provides an important guide to breed [39]. MYB-CC TFs are the members of the MYB TF superfamily which are characterized by containing a conserved MYB DNA-binding domain and a coiled-coil (CC) domain. In this study, *GsMYB10* cloned from wild soybean belongs to the MYB-CC subfamily [30]. Many functions of the MYB-CC family have been reported about Pi availability. Overexpressing *GmPHR25* increased Pi concentration in transgenic soybean hairy roots under normal conditions, accompanied with a significant decrease in hairy root growth, *GmPHR25* is a vital regulator in the P signaling network, and controls Pi homeostasis in soybean [37]. The deficiency of phosphorus (P) and the toxicity of aluminum (Al) are recognized as two major nutritional stress factors that significantly hinder plant growth in acidic soils [40, 41]. Tang et al discovered that the exogenous addition of phosphorus could mitigate aluminum toxicity, indicating that phosphorus has the potential to enhance root morphological development and nutrient uptake in plants under aluminum stress [42]. A total of 35 MYB-CC family members were identified in soybean, and the *GsMYB10* gene (named *GmPH19*) was significantly up-regulated in soybean roots during phosphorus starvation treatment. In this study, the expression of *GsMYB10* was up-regulated under aluminum treatment. The relative root elongation and fresh weight of *GsMYB10* overexpressed plants was significantly greater than that of wild type, meanwhile the Al content was significantly decreased (Fig. 4). Overexpression of wheat *TaALMT1* in barley can improve P absorption and grain yield of acidic soil crops, which is largely attributed to the *TaALMT1*-mediated Al resistance that maintains normal root growth in acidic soil [43]. Whether *GsMYB10* plays the same role in regulating soybean phosphorus homeostasis and the P absorption of the *GsMYB10*-OE plants with Al-treatment remains to be explored.

Plants exposed to aluminum stress will produce toxic effects in roots in a short time. The root growth inhibition is certainly the most easily recognizable trait of Al toxicity which can widely be marked as a measure of Al toxicity in plants. In this study, treatment with AlCl_3_ significantly inhibited the growth of both *GsMYB10*-OE plants and wild-type plants. However, the root growth of the *GsMYB10* overexpression lines was less affected compared to that of the WT plants (Fig. 4 and Supplementary Fig. 3). Hematoxylin-stained root tips were used as indicators of Al tolerance [44]. The shallower hematoxylin staining indicates less apical bound aluminum. As can be seen in Fig. 4A, the overexpression plants of *GsMYB10* were significantly less colored than wild type. In addition, the accumulation of Al^3+^ in the root tips of transgenic plants was significantly lower than that of wild type (Fig. 4B and Fig. 5D). These results indicate that overexpression of *GsMYB10* may help increase the aluminum tolerance of transgenic plants by reducing the accumulation of aluminum ions in roots.

The RNA-seq is an effective method to elucidate the molecular mechanism of gene functions in plant. In present study, transcriptome analysis showed that aluminum treatment induced a large number of DEGs in *GsMYB10-OE* plants compared with those of wild-type plants (Fig. 6A). GO enrichment analysis showed that genes related to plant cell wall modification were significantly enriched. As the first physical barrier of aluminum, cell wall plays an increasingly important role in aluminum tolerance [45]. It is generally believed that the main binding site of aluminum in cell wall is pectin polysaccharide [41]. In this study, the results of transcriptome analysis and qRT-PCR showed that the expression levels of *Glyma.19g006200* and *Glyma.05g005800* in *GsMYB10-OE* plants were significantly increased under aluminum treatment compared with those of wild type (Fig. 6C and D). We found *Glyma.19g006200* and *Glyma.05g005800* encode a cell wall localized polygalacturonase (PG), one of the hydrolases responsible for cell wall pectin degradation [46]. The results suggest that the increased expression of *Glyma.19g006200* and *Glyma.05g005800* may change the composition and structure of pectin and activate cell wall defense against aluminum stress in *GsMYB10-OE* plants. GO enrichment analysis also show that *Glyma.12g080100*, *Glyma.12g101800*, *Glyma.08g093900* and *Glyma.13g322500* belong to the GO:0010411 (xyloglucan metabolic process) were enriched. Recent studies have found that hemicellulose in *Arabidopsis* is as important as pectin in binding Al, and affecting Al tolerance [13]. A subclass of XTH genes encoding xyloglucan endotransglucosylases (XET) responsible for cleaving and rejoining hemicellulosic xyloglucan polymers during cell expansion were revealed to respond to and function in Al stress. *XTH31* transcript levels were selectively reduced by Al stress. *XTH31* is involved in cell wall modification and cell elongation lead to increased aluminum tolerance, and *XTH17* could physically interact with *XTH31* to regulate the Al binding ability of cell wall, thus affecting Al sensitivity [13, 47] (Fig. 6C). Recent study shows that *XTH17* and *ELP* are the potential downstream genes of *WRKY47*, *XTH17* and *ELP* mediate the Al tolerance conferred by *WRKY47* in *Arabidopsis. ANAC017* regulates aluminum resistance in *Arabidopsis* by acting upstream of *XTH31* [48, 49]. *Glyma.12g101800* and *Glyma.13g322500* was the homolog of *AtXTH31*, we found that the expression of *Glyma.12g101800* and *Glyma.13g322500* were increased significantly in *GsMYB10* overexpressing plants. We speculated that *GsMYB10* may also act as the upstream regulator of them. Therefore, *GsMYB10* may enhance resistance to aluminum by regulating the expression of cell wall-related genes.

In addition, *GmNRAMP7* (*Glyma.06g115800*) belongs to the Nramp (Natural resistance-associated macrophage protein) family which were induced in *GsMYB10-OE* plant under Al treatment. *OsNrat1* belonging to the Nramp family and encoding a plasma membrane-localized transporter plays an important role in rice aluminum tolerance by operating in concert with a vacuolar ABC transporter. *Nrat1* can also increase aluminum tolerance in *Arabidopsis* by the same way [50, 51]. Phylogenetic relationship showed that *GmNRAMP7* is the only soybean NRAMP protein in a small branch with several monocot NRAMPs [52]. Moreover, the expression of some vacuolar ABC transporter genes such as *Glyma.13g118800*, *Glyma.20g242000*, *Glyma.08g270300* and *Glyma.08g194600* were increased by Al treatment in *GsMYB10-OE* plant. The results indicate that the *GsMYB10* gene may mitigate the toxic effects of aluminum ions in the roots by regulating the expression of the aforementioned differential genes, thereby enhancing the resistance of *GsMYB10-OE* plants.

In summary, the data presented in this paper indicate that *GsMYB10*, as a new soybean MYB-CC TF, is a positive regulator and increases the adaptability of soybeans to acidic aluminum stress. These findings will contribute to the understanding of soybean response to acidic aluminum stress.

## Conclusions

The *GsMYB10* gene could positively regulate soybean tolerance to acidic aluminum stress. The results suggested that *GsMYB10* may regulate the aluminum resistance of soybean by regulating differential genes related to cell wall components, modification, and aluminum ion transport.

## Methods

### Plant materials and stress treatments

The seeds of wild soybean BW69 line and soybean cultivar HC6 were obtained from the Guangdong Subcenter of the National Center for Soybean Improvement (Guangzhou, China). For hydroponics, seeds were sterilized with 1% (v/v) NaClO for 5 min, subsequently washed three times with deionized water. The seeds were then germinated in vermiculite pots for 4 d in a growth chamber with a 14-h light (28 °C)/10-h dark (24 °C) photoperiod with approximately 100 µM m^− 2^s^− 1^ photon density and 70% relative humidity. The resultant seedlings were then grown in full strength nutrient solution for 24 h before various treatments. For the time-course experiment, soybean seedlings were transplanted to Al (50 µM AlCl_3_, 0.5 mM CaCl_2_, pH4.5) treatments for 0, 1, 2, 4, 8, 12, and 24 h, respectively. For the Al dose experiment, soybean seedlings were treated with 0, 25, 50, 75, and 100 µM AlCl_3_ in 0.5 mM CaCl_2_ solution (pH4.5) for 8 h, respectively [53]. The samples of root tips (0–6 cm) were separately harvested for gene expression assays for the concentration response experiment and time-course experiment. In addition, wild soybean (BW69) and soybean cultivar HC6 was sown in the field of the agricultural trial station of South China Agricultural University. The samples of roots, stems, leaves, flowers, pods and apical tissues were harvested and immediately dipped into liquid nitrogen during the pod stage. The samples were then stored at -80℃. All the experiments had three biological replicates.

### RNA extraction and quantitative real-time PCR analysis

Total RNA was extracted using a Trizol reagent (TIANGEN, China) and treated with DNase I (Takara) to remove genomic DNA. Synthesis of cDNA was conducted using Revert Aid First Strand cDNA synthesis Kit (Takara). Triplicate quantitative assays were performed on cDNA with the SYBR Green Master mix and the SsoFast EvaGreen Supermix Kit (BIO-RAD) on an ABI 7900 sequence detection system according to the manufacturers’ instructions. The data were normalized using the reference gene *Actin3*. The quantitative variation between the examined replicates was evaluated by the 2^–∆∆Ct^ method [54]. The primers used for qRT-PCR were listed in Supplementary Table S1.

### Cloning and sequence analysis

The sequence information of *GsMYB10* was obtained from the database of the National Center for Biotechnology Information (NCBI) with the accession number LOC100781878. To obtain the full-length ORF of *GsMYB10*, the primers (5´-TGGCTTTGCAGGTTGA-3´and 5´-AACTCATATTTGGCTAG-3´) were designed to amplify the *GsMYB10* gene on the basis of soybean genome sequence information. The PCR fragments were cloned into the pLB vector (Zero Background Quick Cloning Kit, TIANGEN, China).

Phylogenetic tree analysis was conducted by a Clustal X multiple-sequence alignment and the Maximum likelihood (ML) method from MEGAX [55]. Phylogenetic analysis of MYB-CC family members in soybean [37], Arabidopsis [32], and rice [35], as previously reported. Multiple sequence alignments were performed using Clustal X software based on the two conserved domains (MYB and coiled-coil) of GsMYB10 and other published MYB-CC family genes. All the sequences of the *GsMYB10* gene were obtained using BLAST in the NCBI and Phytozome database.

### Subcellular localization analysis

To determine the subcellular localization of GsMYB10 protein, the complete open reading frame (ORF) without the stop codon was amplified and inserted into the *Nco*I and *Spe*I sites of pCAMBIA1302 containing the GFP reporter gene under the control of cauliflower mosaic virus (CaMV) 35S promoter to form a construct 35S::GsMYB10-GFP. The fusion construct (35S::GsMYB10-GFP) and control vector (35S::GFP) were integrated into Agrobacterium tumefaciens GV3101. Tobacco (*Nicotiana benthamiana*) leaves were agroinfiltrated with GV3101 carrying either the fusion construct or the control using the method described previously. The infiltrated plants were grown for an additional two days prior to fluorescence signal detection using a laser scanning confocal microscope (Leica TCS SP8).

### Transcriptional activation activity assay

To detect transcriptional activity of GsMYB10 protein, the full-length coding region of *GsMYB10* was amplified with special primers (Supplementary Table S1) and inserted into the sites of *Nco*I and *Bam*HI of pGBKT7 vector to form the GsMYB10-pGBKT7 construct (Clontech). The fusion construct and empty vector were separately transformed in yeast strain Y2H. The yeast cells were plated on synthetic dropout SD/-Trp medium. Activity of α-galactosidase was examined by plating the transformants on SD/-Trp medium containing X-α-Gal. The above experimental methods are analyzed according to previous studies [56].

### Vector construction and plant transformation

The coding sequence of *GsMYB10* was cloned into pZY101 vector to generate overexpression construct. The *GsMYB10*-pZY101 construct was transferred into soybean cultivar Huachun 6 using the transformation method of Agrobacterium-mediated cotyledon node described previously [57]. The transgenic positive plants were identified with specific primers (Supplementary Table 1). Expression levels of *GsMYB10* in transgenic plants were examined by qRT-PCR. The transgenic lines of T_3_ generation were used for the subsequent experiments.

### Phenotype and statistical analyses of transgenic plants treated with aluminum

The soybean seeds of T_3_ generation were sown in pots filled with vermiculite for 3 days. Then the seedlings were transplanted into 0.5 mM CaCl_2_ (pH = 4.5) solution and cultured for 24 h. Pre-cultured seedlings were exposed to 0.5 mM CaCl_2_ solution containing 0 or 25 µM AlCl_3_ (pH4.5). The root length was measured by taking pictures with a Nikon camera and then using the software Image J. The relative root elongation (RRE) was calculated to evaluate Al sensitivity [58]. The fresh weights were measured using a Sartorius BSA224S-CW 1/1000 analytical balance.

Root tips were stained with the hematoxylin which was used as an indicator of Al tolerance [59, 60]. After 8 h of treatment, the root tips of 2 cm length were washed with distilled water for half an hour, stained with hematoxylin for half an hour, and then washed again with distilled water for half an hour. The cleaned root tips were imaged with a Stereomicroscope Leica S8AP0.

For determination of root Al content, the last 0–2 cm root tips from the roots with similar length were washed three times with distilled water. 12 roots were extracted by 2 M HCl for 48 h with occasional shaking. Content of Al^3+^ was determined by inductively coupled plasma-atomic emission spectrometry (ICP-AES, IRIS-Advantage, 710-ES, VARIAN, USA).

### Measurements of MDA and Proline content

For physiological parameter measurements, the leaves of aluminum treated WT and *GsMYB10*-OE plants were harvested for the measurements of MDA content and proline content. MDA content is determined by thiobarbituric acid (TBA) reaction [61]. Proline content is determined by sulfosalicylic acid method [62].

### Transcriptome analysis of transgenic soybean plants

Total RNA was extracted from mock-treated (0 µM AlCl_3_) and Al-treated (25 µM AlCl_3_) for 8 h root of the seedlings (HuaChun6 and *GsMYB10-OE*) using Trizol reagent (Invitrogen, CA, USA). RNA-sequencing (RNA-seq) was performed by LC-BIO (Hangzhou, China). There were three biological replicates for each sample. GO term enrichment analysis of gene sets was performed to identify the enriched GO terms. The calculated P-value was subjected to Bonferroni correction, taking a corrected *p*-value ≦ 0.05 as a threshold for significance. GO terms fulfilling this condition are defined as significantly enriched GO terms in DEGs [63].

### Statistical analyses

All statistical analyses were performed by using IBM SPSS Statistics for Windows, Version 22.0 and Graphpad Prism 8.0.1. Data were represented as mean ± SD (standard deviation) of three biological replicates. Comparison between different groups was tested by one-way ANOVA, followed by *t*-test. A *p*-value less than 0.05 was considered significantly different (*, *P* < 0.05; **, *P* < 0.01).

## Electronic supplementary material

Below is the link to the electronic supplementary material.

Supplementary Material 1

## Data Availability

All data generated in this study are included in this published article (additional files). The RNA-seq data has been uploaded to NCBI database, with accession number PRJNA833532

## Abbreviations

CC: Coiled-coil
TFs: Transcription factors
DEGs: Differentially expressed genes
HC6: The cultivar HuaChun 6
WT: Wild type
MDA: Malondialdehyde
RNA-seq: RNA-sequencing
GO: Gene ontology

## References

[1] von Uexküll HR, Mutert E. Global extent, development and economic impact of acid soils. Plant Soil. 1995;171(1):1–15.

[2] Kochian LV. Cellular mechanisms of aluminum toxicity and resistance in plants. Annu Rev Plant Physiol Plant Mol Biol. 1995;46(1):237–60.

[3] Chauhan DK, Yadav V, Vaculík M, Gassmann W, Pike S, Arif N, Singh VP, Deshmukh R, Sahi S, Tripathi DK. Aluminum toxicity and aluminum stress-induced physiological tolerance responses in higher plants. Crit Rev Biotechnol. 2021;41(5):715–30. 33866893 10.1080/07388551.2021.1874282

[4] Kochian LV, Pineros MA, Liu J, Magalhaes JV. Plant adaptation to acid soils: the molecular basis for crop aluminum resistance. Annu Rev Plant Biol. 2015;66:571–98. 25621514 10.1146/annurev-arplant-043014-114822

[5] Sasaki T, Yamamoto Y, Ezaki B, Katsuhara M, Ahn SJ, Ryan PR, Delhaize E, Matsumoto H. A wheat gene encoding an aluminum-activated malate transporter. Plant J. 2004;37(5):645–53. 14871306 10.1111/j.1365-313x.2003.01991.x

[6] Liu J, Magalhaes JV, Shaff J, Kochian LV. Aluminum-activated citrate and malate transporters from the MATE and ALMT families function independently to confer Arabidopsis aluminum tolerance. Plant J. 2009;57(3):389–99. 18826429 10.1111/j.1365-313X.2008.03696.x

[7] Ryan PR, Raman H, Gupta S, Horst WJ, Delhaize E, Kellogg E, Buell CR. A second mechanism for aluminum resistance in wheat relies on the constitutive efflux of citrate from roots. Plant Physiol. 2009;149(1):340–51. 19005085 10.1104/pp.108.129155 PMC2613747

[8] Yokosho K, Yamaji N, Ma JF. An Al-inducible MATE gene is involved in external detoxification of Al in rice. Plant J. 2011;68(6):1061–69. 10.1111/j.1365-313X.2011.04757.x 21880027

[9] Maron LG, Piñeros MA, Guimarães CT, Magalhaes JV, Pleiman JK, Mao CZ, Shaff J, Belicuas SNJ, Kochian LV. Two functionally distinct members of the MATE (multi-drug and toxic compound extrusion) family of transporters potentially underlie two major aluminum tolerance QTLs in maize. Plant J. 2010;61(5):728–40. 20003133 10.1111/j.1365-313X.2009.04103.x

[10] Hoekenga OA, Maron LG, Piñeros MA, Cançado GMA, Shaff J, Kobayashi Y, et al. *AtALMT1*, which encodes a malate transporter, is identified as one of several genes critical for aluminum tolerance in *Arabidopsis*. Proc Natl Acad Sci. 2006;103(25):9738–43. 16740662 10.1073/pnas.0602868103 PMC1480476

[11] Collins N, Shirley N, Saeed M, Pallotta M, Gustafson J. An *ALMT1* gene cluster controlling aluminum tolerance at the *Alt4* locus of rye (*Secale cereale* L). Genetics. 2008;179(1):669–82. 18493079 10.1534/genetics.107.083451 PMC2390642

[12] Gruber BD, Ryan PR, Richardson AE, Tyerman SD, Ramesh S, Hebb DM, Howitt SM, Delhaize E. *HvALMT1* from barley is involved in the transport of organic anions. J Exp Bot. 2010;61(5):1455–67. 20176888 10.1093/jxb/erq023 PMC2837267

[13] Zhu X, Shi Y, Lei G, Fry S, Zhang B, Zhou Y, et al. XTH31, encoding an in vitro XEH/XET-active enzyme, regulates aluminum sensitivity by modulating in vivo XET action, cell wall xyloglucan content, and aluminum binding capacity in Arabidopsis. Plant Cell. 2012;24(11):4731–47. 23204407 10.1105/tpc.112.106039 PMC3531863

[14] Yamaji N, Chao F, Nagao S, Yano M, Sato Y, Nagamura Y, Jian F. A zinc finger transcription factor *ART1* regulates multiple genes implicated in aluminum tolerance in rice. Plant Cell. 2009;21(10):3339–49. 19880795 10.1105/tpc.109.070771 PMC2782276

[15] Iuchi S, Koyama H, Iuchi A, Kobayashi Y, Kitabayashi S, Kobayashi Y, Ikka T, Hirayama T, Shinozaki K, Kobayashi M. Zinc finger protein *STOP1* is critical for proton tolerance in *Arabidopsis* and coregulates a key gene in aluminum tolerance. Proc Natl Acad Sci U S A. 2007;104(23):9900–5. 17535918 10.1073/pnas.0700117104 PMC1887543

[16] Huang C, Yamaji N, Chen Z, Ma J. A tonoplast-localized half-size ABC transporter is required for internal detoxification of aluminum in rice. Plant J. 2012;69(5):857–67. 22035218 10.1111/j.1365-313X.2011.04837.x

[17] Sawaki Y, Iuchi S, Kobayashi Y, Kobayashi Y, Ikka T, Sakurai N, Fujita M, Shinozaki K, Shibata D, Kobayashi M. *STOP1* regulates multiple genes that protect *Arabidopsis*Protonproton and aluminum toxicities. Plant Physiol. 2009;150(1):281–94. 19321711 10.1104/pp.108.134700 PMC2675709

[18] Yokosho K, Yamaji N, Fujii-Kashino M, Ma JF. Functional analysis of a mate gene *OSFRDL2* revealed its involvement in Al-induced secretion of citrate, but a lower contribution to Al tolerance in rice. Plant Cell Physiol. 2016;57(5):976–85. 26872836 10.1093/pcp/pcw026

[19] Ding Z, Yan J, Xu X, Li G, Zheng S. *WRKY46* functions as a transcriptional repressor of *ALMT1*, regulating aluminum-induced malate secretion in Arabidopsis. Plant J. 2013;76(5):825–35. 24118304 10.1111/tpj.12337

[20] Li G, Wang Z, Yokosho, Ding B, Fan W, Gong Q, et al. Transcription factor *WRKY22* promotes aluminum tolerance via activation of *OsFRDL4* expression and enhancement of citrate secretion in rice (*Oryza sativa*). New Phytol. 2018;219(1):149–62. 29658118 10.1111/nph.15143

[21] Han Z, Wang J, Wang X, Zhang X, Cheng Y, Cai Z, Nian H, Ma Q. *GmWRKY21*, a soybean wrky transcription factor gene, enhances the tolerance to aluminum stress in *Arabidopsis thaliana*. Front Plant Sci. 2022;13:833326. 35958220 10.3389/fpls.2022.833326 PMC9359102

[22] Wang X, Niu Y, Zheng Y. Multiple functions of MYB transcription factors in abiotic stress responses. Int J Mol Sci. 2021;22(11):6125. 34200125 10.3390/ijms22116125 PMC8201141

[23] Seo JS, Sohn HB, Noh K, Jung C, An JH, Donovan CM, Somers DA, Kim DI, Jeong S, Kim C, et al. Expression of the *Arabidopsis AtMYB44* gene confers drought/salt-stress tolerance in transgenic soybean. Mol Breed. 2012;29(3):601–8.

[24] Liu T, Chen T, Kan J, Yao Y, Guo D, Yang Y, Ling X, Wang J, Zhang B. The *GhMYB36* transcription factor confers resistance to biotic and abiotic stress by enhancing *PR1* gene expression in plants. Plant Biotechnol J. 2022;20(4):722–35. 34812570 10.1111/pbi.13751 PMC8989497

[25] He Y, Dong Y, Yang X, Guo D, Qian X, Yan F, Wang Y, Li J, Wang Q. Functional activation of a novel R2R3-MYB protein gene, *GmMYB68*, confers salt-alkali resistance in soybean (*Glycine max* L). Genome. 2020;63(1):13–26. 31550433 10.1139/gen-2018-0132

[26] An J, Li R, Qu F, You C, Wang X, Hao Y. R2R3-MYB transcription factor *MdMYB23* is involved in the cold tolerance and proanthocyanidin accumulation in apple. Plant J. 2018;96(3):562–77. 30054966 10.1111/tpj.14050

[27] Su L, Lv A, Wen W, Fan N, Li J, Gao L, Zhou P, An Y. *MsMYB741* is involved in alfalfa resistance to aluminum stress by regulating flavonoid biosynthesis. Plant J. 2022;112(3):756–71. 36097968 10.1111/tpj.15977

[28] Gao L, Liu X, Gao K, Cui M, Zhu H, Li G, Yan J, Wu Y, Ding Z, Chen XW, Ma J, Harberd NP. *ART1* and putrescine contribute to rice aluminum resistance via *OsMYB30* in cell wall modification. J Integr Plant Biol. 2023;65(4):934–49. 36515424 10.1111/jipb.13429

[29] Wang H, Yin X, Du D, Liang Z, Han Z, Nian H, Ma Q. *GsMYB7* encoding a R2R3-type MYB transcription factor enhances the tolerance to aluminum stress in soybean (*Glycine max* L). BMC Genomics. 2022;23(1):529. 35869448 10.1186/s12864-022-08744-w PMC9306046

[30] Rubio V, Linhares F, Solano R, Martin AC, Iglesias J, Leyva A, Paz-Ares J. A conserved MYB transcription factor involved in phosphate starvation signaling both in vascular plants and in unicellular algae. Genes Dev. 2001;15(16):2122–33. 11511543 10.1101/gad.204401 PMC312755

[31] Nilsson L, Muller R, Nielsen TH. Increased expression of the MYB-related transcription factor, PHR1, leads to enhanced phosphate uptake in *Arabidopsis thaliana*. Plant Cell Environ. 2007;30(12):1499–512. 17927693 10.1111/j.1365-3040.2007.01734.x

[32] Sun L, Song L, Zhang Y, Zheng Z, Liu D. *Arabidopsis PHL2* and *PHR1* act redundantly as the key components of the central regulatory system controlling transcriptional responses to phosphate starvation. Plant Physiol. 2016;170(1):499–514. 26586833 10.1104/pp.15.01336 PMC4704584

[33] Guo M, Ruan W, Li C, Huang F, Zeng M, Liu Y, Yu Y, Ding X, Wu Y, Wu Z, Mao C, Yi K, Wu P, Mo X. Integrative comparison of the role of the phosphate response1 subfamily in phosphate signaling and homeostasis in rice. Plant Physiol. 2015;168(4):1762–76. 26082401 10.1104/pp.15.00736 PMC4528768

[34] Zhou J, Jiao F, Wu Z, Li Y, Wang X, He X, Zhong W, Wu P. *OsPHR2* is involved in phosphate-starvation signaling and excessive phosphate accumulation in shoots of plants. Plant Physiol. 2008;146(4):1673–86. 18263782 10.1104/pp.107.111443 PMC2287342

[35] Ruan W, Guo M, Wu P, Yi K. Phosphate starvation induced *OsPHR4* mediates Pi-signaling and homeostasis in rice. Plant Mol Biol. 2017;93(3):327–40. 27878661 10.1007/s11103-016-0564-6

[36] Li X, Wang Y, WU B, Kong Y, LI W, Chang W, Zhang C. *GmPHR1*, a novel homolog of the *AtPHR1* transcription factor, plays a role in plant tolerance to phosphate starvation. J Integr Agric. 2014;13(12):2584–93.

[37] Xue Y, Xiao B, Zhu S, Mo X, Liang C, Tian J, et al. *GmPHR25*, a GmPHR member up-regulated by phosphate starvation, controls phosphate homeostasis in soybean. J Exp Bot. 2017;68(17):4951–67. 28992334 10.1093/jxb/erx292 PMC5853305

[38] Zeng Q, Yang C, Ma Q, Li X, Dong W, Nian H. Identification of wild soybean miRNAs and their target genes responsive to aluminum stress. BMC Plant Biol. 2012;12:182. 23040172 10.1186/1471-2229-12-182 PMC3519564

[39] Ryan PR, Kochian LV. Interaction between aluminum toxicity and calcium uptake at the root apex in near-isogenic lines of wheat (*Triticum Aestivum* L.) differing in aluminum tolerance. Plant Physiol. 1993;102(3):975–82. 12231883 10.1104/pp.102.3.975 PMC158871

[40] Zheng SJ. Crop production on acidic soils: overcoming aluminium toxicity and phosphorus deficiency. Ann Bot. 2010;106(1):183–4. 20570831 10.1093/aob/mcq134 PMC2889811

[41] Kochian LV, Hoekenga OA, Pineros MA. How do crop plants tolerate acid soils? Mechanisms of aluminum tolerance and phosphorous efficiency. Annu Rev Plant Biol. 2004;55:459–93. 15377228 10.1146/annurev.arplant.55.031903.141655

[42] Tan K, Keltjens WG. Interaction between aluminium and phosphorus in sorghum plants. Plant Soil. 1990;124(1):25–32.

[43] Delhaize E, Taylor P, Hocking PJ, Simpson RJ, Ryan PR, Richardson AE. Transgenic barley (*Hordeum vulgare* L.) expressing the wheat aluminium resistance gene (*TaALMT1*) shows enhanced phosphorus nutrition and grain production when grown on an acid soil. Plant Biotechnol J. 2009;7(5):391–400. 19490502 10.1111/j.1467-7652.2009.00403.x

[44] Polle E, Konzak CF, Kattrick JA. Visual detection of aluminum tolerance levels in wheat by hematoxylin staining of seedling roots. Crop Sci. 1978;18(5):823–7.

[45] Yang J, Zhu X, Peng Y, Zheng C, Li G, Liu Y, Shi Z, Zheng S. Cell wall hemicellulose contributes significantly to aluminum adsorption and root growth in *Arabidopsis*. Plant Physiol. 2011;155(4):1885–92. 21285327 10.1104/pp.111.172221 PMC3091086

[46] Cao Y, Zhang Y, Chen Y, Yu N, Liaqat S, Wu W, et al. *OsPG1* encodes a polygalacturonase that determines cell wall architecture and affects resistance to bacterial blight pathogen in rice. Rice. 2021;14(1):36. 33881659 10.1186/s12284-021-00478-9 PMC8060378

[47] Zhu X, Wan J, Sun Y, Shi Y, Braam J, Li G, Zheng S. Xyloglucan endotransglucosylase-hydrolase17 interacts with xyloglucan endotransglucosylase-hydrolase31 to confer xyloglucan endotransglucosylase action and affect aluminum sensitivity in *Arabidopsis*. Plant Physiol. 2014;165(4):1566–74. 24948835 10.1104/pp.114.243790 PMC4119039

[48] Li C, Yan J, Ren J, Sun L, Xu C, Li G, Ding Z, Zheng S. A WRKY transcription factor confers aluminum tolerance via regulation of cell wall modifying genes. J Integr Plant Biol. 2020;62(8):1176–92. 31729146 10.1111/jipb.12888

[49] Tao Y, Wan J, Liu Y, Yang X, Shen R, Zhu X. The NAC transcription factor *ANAC017* regulates aluminum tolerance by regulating the cell wall-modifying genes. Plant Physiol 2022:c197. 10.1093/plphys/kiac197 PMC9342997 35512200

[50] Li J, Liu J, Dong D, Jia X, McCouch SR, Kochian LV. Natural variation underlies alterations in Nramp aluminum transporter (*NRAT1*) expression and function that play a key role in rice aluminum tolerance. Proc Natl Acad Sci. 2014;111(17):6503–6508. 10.1073/pnas.1318975111 PMC4035919 24728832

[51] Xia J, Yamaji N, Kasai T, Ma J. Plasma membrane-localized transporter for aluminum in rice. Proc Natl Acad Sci. 2010;107(43):18381–5. 20937890 10.1073/pnas.1004949107 PMC2972927

[52] Qin L, Han P, Chen L, Walk TC, Li Y, Hu X, Xie L, Liao H, Liao X. Genome-wide identification and expression analysis of NRAMP family genes in soybean (*Glycine Max* L). Front Plant Sci. 2017;8:1436. 28868061 10.3389/fpls.2017.01436 PMC5563376

[53] Wang X, Cheng Y, Yang C, Yang C, Mu Y, Xia Q, Ma Q. QTL mapping for aluminum tolerance in RIL population of soybean (*Glycine max L*.) by RAD sequencing. PLoS ONE. 2019;14(10):e223674. 10.1371/journal.pone.0223674 PMC6818782 31661499

[54] Livak KJ. TD Schmittgen 2001 Analysis of relative gene expression data using real-time quantitative PCR and the 2(-Delta Delta C(T)) Method. Methods.2001; 25(4): 402–8. 11846609 10.1006/meth.2001.1262

[55] Kumar S, Stecher G, Li M, Knyaz C, Tamura K. MEGA X: molecular evolutionary genetics analysis across computing platforms. Mol Biol Evol. 2018;35(6):1547–9. 29722887 10.1093/molbev/msy096 PMC5967553

[56] Ma Q, Xia Z, Cai Z, Li L, Cheng Y, Liu J, Nian H. *GmWRKY16* enhances drought and salt tolerance through an aba-mediated pathway in *Arabidopsis thaliana*. *Front Plant Sci* 2018, 9:1979. 10.3389/fpls.2018.01979 PMC6357947 30740122

[57] Zeng P, Vadnais DA, Zhang Z, Polacco JC. Refined glufosinate selection in *Agrobacterium*-mediated transformation of soybean [*Glycine max* (L.) Merrill]. Plant Cell Rep. 2004;22(7):478–82. 15034747 10.1007/s00299-003-0712-8

[58] Ryan PR, Delhaize E, Randall PJ. Malate efflux from root apices and tolerance to aluminium are highly correlated in wheat. Funct Plant Biol. 1995;22(22):531–6.

[59] Delhaize E, Craig S, Beaton CD, Bennet RJ, Jagadish VC, Randall PJ. Aluminum tolerance in wheat (*Triticum aestivum* L.) (I. uptake and distribution of aluminum in root apices). Plant Physiol. 1993;103(3):685–93. 12231972 10.1104/pp.103.3.685 PMC159037

[60] Liang C, Piñeros MA, Tian J, Yao Z, Sun L, Liu J, Shaff J, Coluccio A, Kochian LV, Liao H. Low pH, aluminum, and phosphorus coordinately regulate malate exudation through *GmALMT1* to improve soybean adaptation to acid soils. Plant Physiol. 2013;161(3):1347–61. 23341359 10.1104/pp.112.208934 PMC3585601

[61] Schmedes A, Hølmer G. A new thiobarbituric acid (TBA) method for determining free malondialdehyde (MDA) and hydroperoxides selectively as a measure of lipid peroxidation. J Am Oil Chem Soc. 1989;66(6):813–7.

[62] Abraham E, Hourton-Cabassa C, Erdei L, Szabados L. Methods for determination of proline in plants. Methods Mol Biol. 2010;639:317–31. 20387056 10.1007/978-1-60761-702-0_20

[63] Tian T, Liu Y, Yan H, You Q, Yi X, Du Z, Xu W, Su Z. agriGO v2.0: a GO analysis toolkit for the agricultural community, 2017 update. Nucleic Acids Res. 2017;45(W1):W122–9. 28472432 10.1093/nar/gkx382 PMC5793732

